# Shaping the light of VCSELs through cavity geometry design

**DOI:** 10.1038/s41377-025-01996-7

**Published:** 2025-09-28

**Authors:** Hang Lu, Omar Alkhazragi, Heming Lin, Tien Khee Ng, Boon S. Ooi

**Affiliations:** 1 Photonics Laboratory, Electrical and Computer Engineering Program, Division of Computer, Electrical, and Mathematical Sciences and Engineering (CEMSE), King Abdullah University of Science and Technology (KAUST), Thuwal, 23955-6900 Kingdom of Saudi Arabia; 2 Department of Electrical Engineering, King Fahd University of Petroleum and Minerals, Dhahran, Saudi Arabia; 3 Department of Electrical, Computer, and Systems Engineering, Rensselaer Polytechnic Institute, Troy, NY, USA

**Keywords:** Semiconductor lasers, Solid-state lasers

## Abstract

Vertical-cavity surface-emitting lasers (VCSELs) are essential in modern optoelectronic systems, driving applications in high-speed optical communications, 3D sensing, and LiDAR. While significant progress has been made in improving VCSEL performance, the role of cavity geometry in optimizing key optical characteristics remains insufficiently explored. This study systematically examines how distinct cavity geometries—circular, square, D-shaped, mushroom-shaped, and pentagonal—affect both the static and dynamic properties of broad-area VCSELs. We analyze their effects on optical power, multimode behavior, beam profile, spatial coherence, and polarization dynamics. Our results show that breaking the continuous rotational symmetry of the cavity effectively increases gain utilization and power, changes the multimode lasing characteristics, shapes the beam, and modifies the polarization. Notably, the pentagonal VCSEL exhibits more than twice the optical power density of its circular counterpart. It also supports the highest number of modes and the fastest mode dynamics, driven by strong mode interaction. These properties make it a strong candidate for high-speed entropy generation. Mushroom-shaped VCSELs demonstrate high power and low spatial coherence, making them ideal for speckle-free imaging and illumination applications. Meanwhile, D-shaped VCSELs provide the most stable polarization and controllable multimode behavior with high power, showcasing their potential for applications that require stable and low-coherence light sources. This study offers a comprehensive analysis of the impact of cavity geometry on VCSEL performance, which provides insights for optimizing VCSEL designs tailored to diverse applications that require distinct properties with broad applicability to advanced imaging, sensing, optical coherence tomography, high-speed communication, and other photonic technologies.

## Abstract (web summary)

Geometry-engineered VCSELs emit tailored multimode light, enabling control over power, coherence, and polarization for advanced photonic functions.

## Graphical abstract

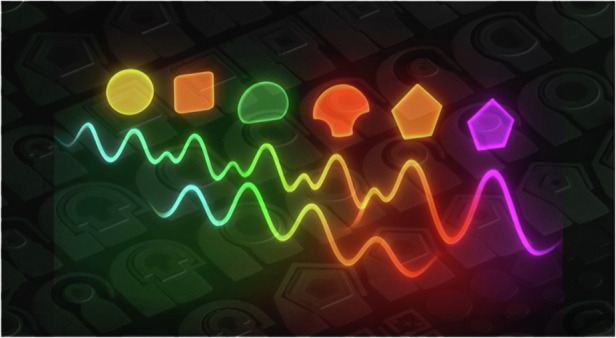

## Introduction

Vertical-cavity surface-emitting lasers (VCSELs) have become a foundational technology in modern optoelectronics, playing a pivotal role in applications in high-speed optical communications, 3D sensing, and LiDAR^1,2^. The widespread use of VCSELs is driven by their unique advantages, including low threshold currents, high efficiency, circular beam profiles, and the ability to be fabricated in large arrays on a single wafer. These qualities have made VCSELs indispensable in applications where scalability, cost-effectiveness, and small footprint are critical. Historically, VCSEL development has focused primarily on single-mode designs due to their high beam quality and polarization stability, well-suited for precision applications in short-range communication and sensing^3–5^. Therefore, conventional single-mode, small-aperture (<5 µm) VCSELs with circular geometries meet requirements in applications where high coherence and low-energy consumption are key. However, they encounter challenges in providing high power due to the small scale of the gain medium in the few-micrometer aperture. The simplest solution is using broad-area VCSELs, which are proposed as a promising alternative with higher power and multiple functionalities^6–11^.

However, most broad-area VCSELs adopt circular cavities, which inherently support whispering gallery modes (WGMs) due to their continuous rotational symmetry, which causes the optical field to undergo total internal reflection along the curved boundary of the aperture^8,9,12,13^. This results in a ring-like optical intensity distribution where light is confined near the cavity edge, leaving the central gain region underutilized, thereby reducing gain utilization efficiency and ultimately limiting the achievable output power. Furthermore, the power cannot be increased arbitrarily with the increase of the aperture size due to insufficient current injection and poor thermal dissipation^14^. In addition to the low power, traditional circular cavities tend to meet issues related to strong mode competition and polarization instability under high-power operation, restricting their utility in applications requiring both stable and high-power light sources. The scope of optoelectronic applications expands with the increasing demands on VCSEL technology. The conventional broad-area VCSELs face limitations in broader applications that necessitate not only the increasing output power, but also the customized spatial coherence, controllable multimode lasing, and tuning polarization dynamics. Solving these challenges unlocks the VCSELs in a large range of applications, such as high-speed optical wireless communication, speckle-free imaging and illumination, parallel integrated sensing, and multi-channel high-speed random number generation^14,15^, which emphasizes the need to enhance the power, as well as optimize both the static and dynamic multimode lasing properties of the light source.

Modifying the cavity shape presents a promising solution to these challenges as it adds another degree of freedom to control the lasing behavior without additional cost. By reshaping the boundary condition of the light field, it is possible to enhance optical power while simultaneously tuning the coherence and multimode lasing dynamics. For example, the D-cavity is proposed to stabilize the lasing emission of edge-emitting lasers^16^, and a violin-shaped cavity has been demonstrated for ultrafast random number generation, harnessing its ultrafast dynamics^17^. Despite the potential of non-symmetric cavities to reshape the laser performance, most of the existing research has only focused on edge-emitting lasers instead of VCSELs^18–21^. Compared to VCSELs, the deformed cavity in the edge-emitting laser causes a problem in the low light collection efficiency since it lacks directional emission with the curved boundary. Furthermore, the edge-emitting configurations have a large footprint and cannot form a 2D array, limiting their use in consumer electronics and other low-energy cost applications. Although a few works on chaotic cavities VCSELs have been reported in recent years^8,22–24^; and even in organic photonic platforms^25^, they only focus on a single cavity and specific characteristics. A comprehensive study that compares different geometries across various performance metrics on VCSEL remains lacking, leaving open questions about how cavity shape impacts the broader interplay between optical power, multimode lasing behavior, spatial coherence, and polarization dynamics.

This study aims to address these gaps by systematically examining five distinct VCSEL cavity geometries—circular, square, D-shaped, mushroom-shaped, and pentagonal—and their effects on both static and dynamic performance metrics. In this work, we first present a comparison of these five VCSEL cavity geometries, investigating static properties such as optical power, spectral characteristics, spatial coherence, polarization suppression, as well as multimode lasing dynamics. Our findings reveal that through careful cavity design, broad-area VCSELs can achieve significant improvements in optical power, controllable coherence, customized multimode lasing, and modified polarization stability. Notably, pentagonal VCSELs achieve the highest improvement (103%) in the optical power output, which is more than double of the traditional circular VCSEL, along with the fastest polarization dynamics due to strong mode interaction, which opens its opportunity as a high-entropy source for high-speed random number generation and reservoir computing^26–28^. In contrast, the D-shaped VCSELs show the most stable polarization and controllable multimode lasing with high power, exhibiting their promise in applications that require stable and low-coherence light sources. Moreover, the mushroom-shaped VCSELs demonstrate both high optical power and low spatial coherence, making them well-suited for imaging and illumination applications. By detailing the influence of different cavity geometries on VCSEL performance, these insights contribute to the design and optimization of next-generation VCSELs that meet the diverse and evolving needs of modern optoelectronic applications, with no extra manufacturing cost to the existing VCSEL technology.

## Results

### Device fabrication and optical power characterization

**Fig. 1:**
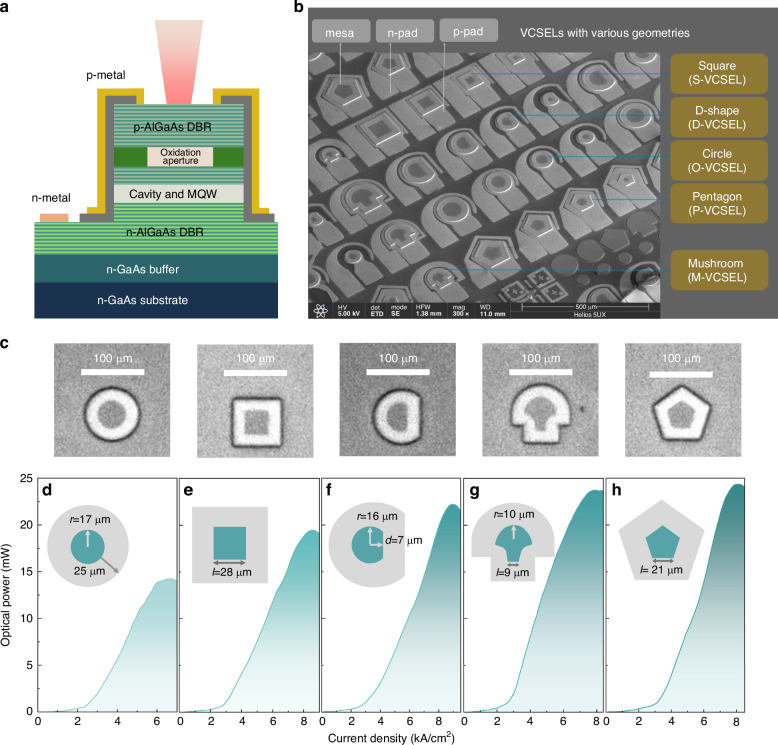
**Schematic of VCSELs and optical power measurement**. **a** Schematic cross-sectional view of a 940-nm VCSEL wafer. **b** Scanning electron microscope (SEM) image of the VCSELs with five distinct cavity geometries: square (S-VCSEL), D-shaped (D-VCSEL), circular (O-VCSEL), pentagonal (P-VCSEL), and mushroom-shaped (M-VCSEL). **c** top-view images captured after oxidation process of each VCSEL. **d**–**h** Light-current density (L-J) characteristics of VCSELs with varying cavity geometries from the same cell. The insets display the respective geometrical designs

To investigate the impact of cavity geometry on VCSELs, groups of VCSELs with varying shapes were fabricated using a commercial 940-nm VCSEL wafer. The schematic of VCSELs and the wafer information are shown in Fig. 1a. The fabrication followed the standard steps, which are similar to our previous work^14^. Figure 1b presents a scanning electron microscope (SEM) image of the fabricated VCSELs, each with a different cavity geometry. The distinct shapes—square, D-shaped, circular, pentagonal, and mushroom-shaped—are labeled on the right, and the structures, including mesas, n-pad, and p-pad, are labeled on the top. In addition, the detailed view of the aperture after the oxidation is shown in the Fig. 1c captured using a near-infrared microscope, where the oxidation process was designed to stop when the oxidation depth reached 25 µm from the edge of the mesa to the aperture. To maintain consistency in material properties and processing conditions, the results shown in the following measurements are from the VCSELs fabricated within the same cell.

**Fig. 2:**
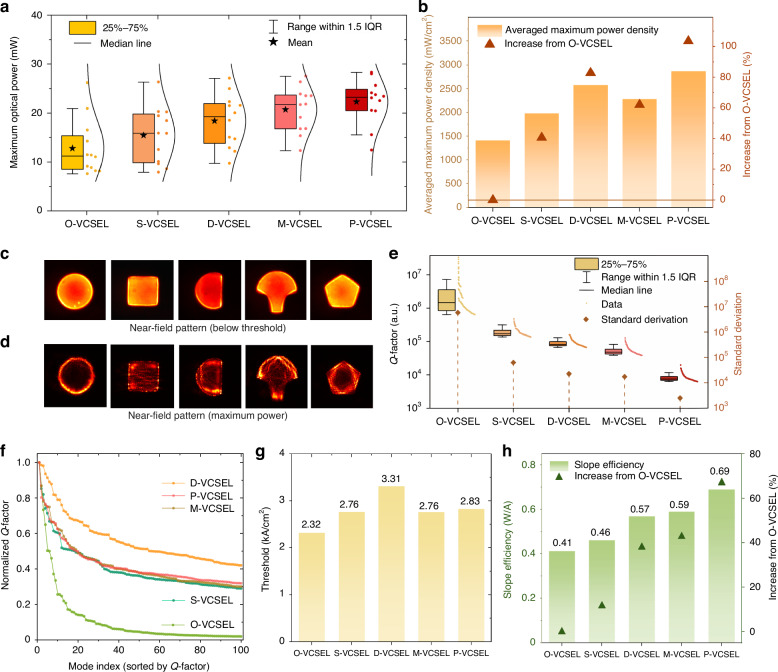
**Electrical and optical characterization of VCSELs with different cavities**. **a** Distribution of maximum optical power for different VCSEL geometries across 12 devices for each shape, with the mean (star) and median (solid line) indicated. **b** Bar chart comparing the averaged optical power density (mW/cm²) across the emission areas of the five cavity geometries, normalized to the optical power density of the circular (O-VCSEL) design. **c** Near-field patterns below the lasing threshold. **d** Near-field patterns at maximum power. **e** Simulated Q-factor distributions for the top 100 optical modes of each geometry with the standard deviation of each geometry. **f** Normalized Q-factors of the top 100 modes for each VCSEL geometry, sorted by decreasing Q-factor. **g** Averaged threshold current density for each geometry. **h** Averaged slope efficiency for each geometry, and the increase compared to the O-VCSEL

Firstly, we measure the optical power characteristic of VCSELs with five distinct cavity shapes, as shown in Fig. 1d–h, with the schematic representation of the cavity shape with key parameters. These devices were measured from the same local cell (i.e., spatially adjacent region on the wafer) to ensure minimal variation from growth and fabrication processes. Starting with the traditional circular VCSEL (O-VCSEL) shown in Fig. 1d, the device has an aperture radius of 17 µm and exhibits the lowest optical power among all the geometries, reaching a maximum power of 14 mW before thermal roll-off. This geometry also reaches saturation at a relatively low current, reflecting its limitations in optical power scalability. The square VCSEL (S-VCSEL), shown in Fig. 1e with an aperture side length of 28 µm, achieves a higher optical power output than the circular design, indicating an improvement in power efficiency with this shape, but it is still lower than other cavities without continuous rotational symmetry. For example, the D-shaped VCSEL (D-VCSEL), presented in Fig. 1f, has a curved radius of 18 µm and a flat-to-center distance of 7 µm. This geometry surpasses both the circular and square designs in terms of optical power, which is consistent with previous findings^8^. Figure 1g shows the mushroom-shaped VCSEL (M-VCSEL), which combines a semicircular top section with a narrower square “stem”. Due to the nonuniformity in the oxidation process, the stem shows slight deformation in the curves. This unique configuration enables the M-VCSEL to achieve the second-highest optical power of 23.5 mW. Finally, the pentagonal VCSEL (P-VCSEL), with a side length of 21 µm, achieves the highest optical power output at 24.5 mW, as shown in Fig. 1h, demonstrating its superior performance in delivering higher power from a single device. Figure 2a, b expand these results by analyzing the distribution and consistency of power output, as well as the relative power efficiency of each geometry.

Figure 2a presents a box plot showing the maximum optical power achievable by each geometry, with data collected from 12 devices per shape to account for variations within each geometry due to fabrication processes. Each box plot illustrates the interquartile range (25%–75%), providing a measure of data spread, with the median value indicated by a solid black line within each box. A star marks the mean value of the maximum power across all the measured devices in each geometry. In these configurations, the O-VCSEL has the lowest average value (12.78 mW), indicating that traditional circular cavities are limited in terms of high optical power. In contrast, the P-VCSEL achieves the highest maximum power (22.3 mW) with the narrowest spread. The high value indicates the strong power-delivering capability of the pentagonal shape design, while the low variability may suggest less sensitivity to fabrication differences. Furthermore, the M-VCSELs and D-VCSELs also demonstrate relatively high-power outputs compared to conventional O- and S-VCSELs, with median values approaching those of the pentagonal design but with larger variability, which could be caused by the vulnerability to the fabrication process.

Below each box plot, near-field emission patterns are shown in Fig. 2c for each geometry when the devices operate below the lasing threshold. These near fields are used to estimate the effective emission area for each VCSEL. The estimated emission aperture areas of O-, S-, D-, M-, and P-VCSELs are 907 μm², 778 μm², 713 μm², 907 μm², and 778 μm², respectively. Therefore, the power density over the emission area can be calculated. As shown in Fig. 2b, it compares the average highest achievable optical power density over the corresponding emission area for each geometry, and the power density is further normalized to that of the O-VCSEL for better comparison. This figure shows that non-circular geometries exhibit significant improvements in power density over the circular design. Notably, the P-VCSEL achieves the highest power density, which is 2867 mW/cm^2^, representing a 103% increase over the circular VCSEL (1409 mW/cm^2^), indicating a highly efficient use of the gain medium. Moreover, the D-VCSEL and M-VCSEL also show considerable improvement in power density, which is 80% and 60%, respectively, whereas the S-VCSEL demonstrates a moderate increase in power density (40%). These results confirm that by breaking the rotational symmetry of the cavity, the power can be enhanced significantly^8,13^.

One of the critical aspects to explain the power characteristics is revealed by Fig. 2d with the near-field pattern (NFP) across various cavity geometries when they are delivering the highest power, which directly reflects the different degrees of utilization of the gain medium, showing distinct ray dynamics that arise from the geometric constraints of each cavity shape. In conventional O-VCSELs, the structure supports WGMs with high quality factors (Q-factors), which predominantly occupy the periphery of the cavity and lase first, preventing other modes from lasing through mode competition^8,12^. As a result, a significant portion of the gain medium in the central area remains underutilized, limiting the final optical power output and efficiency of the O-VCSEL. In contrast, non-circular geometries exhibit more evenly distributed modes that extend across a broader region of the gain medium, which enhances gain utilization by allowing optical modes to access a larger portion of the cavity, resulting in higher optical power, corresponding to the previous power measurements. The improved gain medium utilization seen in non-circular geometries offers significant advantages for applications requiring high power and suggests an effective strategy for enhancing VCSEL power. Notably, in S-VCSEL and the stem of the M-VCSEL, the NFP shows modes in stripes, which is similar to the Fabry–Perot lasers and is consistent with the observation in a previous work^23^.

To gain deeper insights, we performed numerical simulations of the Q-factor distributions for these geometries under passive conditions using COMSOL Multiphysics, where a 3D simulation model was developed. Constrained by computational resources, we adopted a representative cavity size for simulation. The O-VCSEL was set with a 5 μm radius, while other cavity shapes were adjusted to preserve similar aperture areas. These passive-mode simulations focus on eigenmode distributions, with Q-factors extracted for the top 100 modes in each geometry. Figure 2e illustrates the Q-factor distributions for the top 100 modes of each cavity geometry with their corresponding standard deviation, and Fig. 2f further shows the normalized Q-factors to the maximum value in each case. The results indicate that the O-VCSEL exhibits a steeply declining Q-factor profile, where only a few WGMs have exceptionally high Q-factors. These high-Q modes are likely to reach the lasing threshold at lower injection currents and rapidly consume the available energy, thereby suppressing other modes with low Q-factors and high energy requirements from lasing. This trend is consistent with experimental observations in Fig. 2g, where the O-VCSEL shows the lowest threshold current density (2.32 kA/cm²), reflecting efficient lasing initiation driven by a few dominant modes with minimal energy input. The standard deviation values, also shown in Fig. 2e, quantitatively reflect that O-VCSEL has the highest deviation, while P-VCSELs show a much lower deviation of Q-values. This statistical uniformity supports efficient multimode lasing, as a larger number of modes can be excited under similar injection conditions in P-VCSEL.

From a macroscopic perspective, by modifying the boundary condition of eigenmodes by reshaping the cavity, the gain can be accessed more evenly across both spatial and spectral domains. Mode suppression is significantly reduced since they do not directly share the same gain resource. This enables a regime of inherent multimode coexistence in the chaotic-cavity VCSELs. For example, in Fig. 2f, the D-VCSEL exhibits a relatively uniform Q-factor distribution, enabling the simultaneous excitation of multiple modes under the same injection conditions. This corresponds with the highest measured threshold current density (3.31 kA/cm²) in Fig. 2g, and a “soft” turn-on behavior observed in Fig. 1f. The uniform Q-factor landscape means that the D-VCSEL continually requires energy to support multiple modes one by one. These modes may vary in spatial and spectral properties, increasing the chances of utilizing a broader portion of the gain region, both spectrally and spatially. This behavior also contributes to higher final output power. The S-, M-, and P-VCSELs exhibit intermediate characteristics. The S-VCSEL shows notable Q-factor variation within the first 16 modes, while the P-VCSEL maintains a more uniform and higher Q-factor distribution beyond 40 modes. These differences result in comparable threshold current densities (2.7–2.8 kA/cm²) for these three geometries, consistent with their moderate Q-factor distributions.

The slope efficiencies of the five geometries, shown in Fig. 2h, provide further insights into their energy conversion efficiency above the lasing threshold. The O-VCSEL has the lowest slope efficiency (0.41 W/A), reflecting its limitation in multimode lasing and photon escape efficiency. Despite its low threshold, the circular geometry suffers from inefficient energy conversion as more modes are suppressed. The S-VCSEL achieves a modest improvement in slope efficiency (0.46 W/A). Corresponding to the power characteristics, the D-VCSEL and M-VCSEL demonstrate significantly higher slope efficiencies (0.57 W/A and 0.59 W/A, respectively), and the P-VCSEL exhibits the highest slope efficiency of 0.69 W/A, which represents a 68% increase compared to the conventional O-VCSEL. It is noted that although the D-VCSEL has the most uniform Q-factor distribution, the pentagonal VCSEL achieves the highest power and the highest slope efficiency, suggesting that additional factors, such as reduced diffraction losses and better modal overlap within the gain medium, could contribute to the pentagonal VCSEL’s outperformance. It is also important to note that the Q-factor simulations were conducted under passive conditions, without incorporating current injection and thermal effects, which significantly influence laser behavior under actual operating conditions. As such, the results are intended to provide qualitative guidance on how cavity geometry impacts multimode lasing potential, rather than serving as definitive predictors of real lasing performance.

These results collectively confirm that geometry-specific structural characteristics, such as aperture size and edge configuration, are critical in determining the optical and electrical performance of each VCSEL. The variation in power output and slope efficiency across different geometries highlights the potential of using non-circular geometries, particularly pentagonal designs, to achieve higher power densities and high energy efficiency, offering advantages for applications requiring compact, high-intensity light sources.

### Spectra and beam profile characteristics

**Fig. 3:**
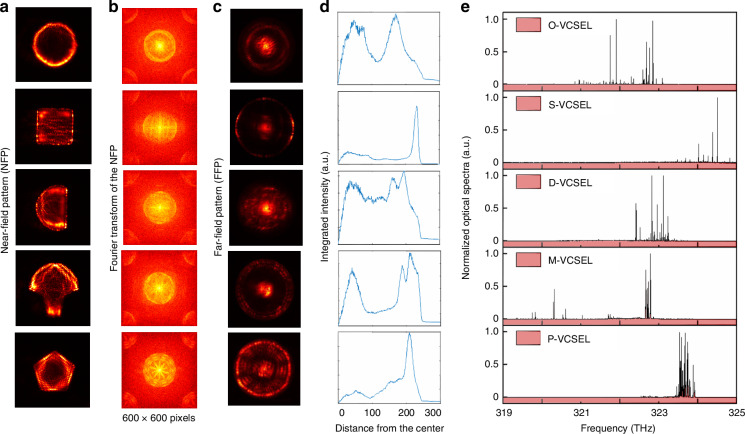
**Beam profile and spectra of VCSELs with different cavity geometries at maximum power output**. **a** Near-field patterns (NFP), showing the light distribution in the cavities. **b** Fourier transforms of the NFPs, representing the spatial frequency components of each beam profile. **c** Far-field patterns (FFP), illustrating beam shape at maximum power. **d** Radial intensity profiles of the FFPs, showing integrated intensity as a function of distance from the beam center. **e** Normalized optical spectra of each geometry, revealing distinct spectral features at maximum power

The influence of cavity geometry extends beyond the optical power, impacting also on the multimode structure, beam profile, and spectral distribution of VCSELs. To develop a deeper understanding of how geometry shapes these emission characteristics, we conducted an analysis of the spectrum, near-field, and far-field profiled of the VCSELs when they operate at the maximum power, as illustrated in Fig. 3. To further explore how the spatial modes distribution translates to beam properties, we examine the Fourier transforms of the NFPs in Fig. 3b alongside the far-field patterns (FFPs) in Fig. 3c. The experimental details of the measurement are provided in the Supplementary materials. Theoretically, the FFP of a light source should closely resemble the Fourier transform of its NFP, as the far-field represents the angular distribution of emitted light^29^. This relationship is well-demonstrated in our results, where the FFPs for each cavity geometry align closely with the Fourier transforms of their corresponding NFPs. In all the VCSELs, the Fourier transforms and the FFPs show a well-defined central region with high-frequency components, indicating a combination of low-order and high-order modes at the highest power. However, the O-VCSEL shows a relatively high coherence represented by the bright center part and the minimal high-frequency components, which exhibit spatially restricted emission caused by its WGM-dominated mode structure. This coherence reflects the O-VCSEL’s confined mode structure, albeit with the drawback of limited gain utilization due to WGMs. In contrast, the more complex shapes, such as the S-, M-, and P-VCSEL geometries, exhibit FFPs with pronounced high-frequency components, whereas the P-VCSEL shows the strongest high-frequency component.

**Fig. 4:**
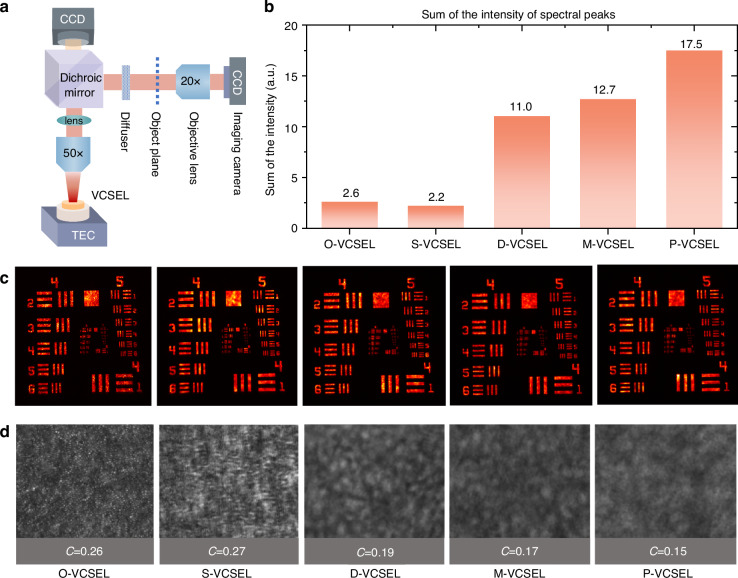
**Spatial coherence analysis of VCSELs with different cavities and their speckles**. **a** Experimental setup for imaging performance assessment, using a 50× objective lens, diffuser, dichroic mirror, and charge-coupled device (CCD) imaging camera to capture images of a standard resolution target and speckle illuminated by each VCSEL. **b** Sum of the normalized intensity peaks in the emission spectra for each VCSEL geometry. **c** Imaging patterns of a resolution target under VCSEL illumination, showing the varying levels of speckle for each geometry. **d** Contrast (C) values calculated from the speckle patterns, indicating spatial coherence levels for each geometry

Additionally, Fig. 3d, e collectively illustrate the relationship between the far field and the spectral characteristics of each VCSEL geometry. The radial intensity profiles in Fig. 3d show the sum of the intensity values of the pixels with the same distance from the center of the FFPs, and the plots in Fig. 3e are the spectra recorded from each VCSEL. Since VCSELs emit light in a single longitudinal mode, modes at higher frequencies (longer angular wave vectors and shorter wavelengths) are emitted at larger angles than those at lower frequencies. Therefore, the profile in Fig. 3d is seen as an analog to the optical spectra shown in Fig. 3e. Among the geometries, the O-VCSEL exhibits a spectral width with two distinct groups in its optical spectrum, which corresponds to its low-order and high-order modes components. In comparison, the S-VCSEL displays a narrower spectrum than the O-VCSEL, indicating its low number of modes. In contrast, the D-, M-, and P-VCSELs exhibit a significantly large number of peaks in the spectra, reflecting more modes existing together in the cavity, beneficial for applications that leverage reduced coherence or expanded spectral coverage, such as speckle-free imaging systems or multi-channel wide-bandwidth communications. Furthermore, we extend the examination to assess the imaging performance and speckle characteristics of VCSELs with various cavity geometries at their maximum power outputs using the setup shown in Fig. 4a, which provides insights into how the spatial coherence of VCSELs affects image clarity and speckle formation, factors critical to applications requiring controlled coherence and speckle-reduced illumination. The imaging performance is strongly influenced by the number of incoherent modes of the light source.

While precisely determining the number of modes in highly multimode lasers is often hindered by spectral overlap^30,31^, we approximate the modal richness by summing the intensities of normalized spectral peaks in Fig. 3e. The aggregate values shown in Fig. 4b serve as a qualitative indicator of the number of active lasing modes and their relative contributions. A higher summed intensity suggests either a larger number of distinct modes, stronger modal amplitudes, or both. This metric is particularly relevant for assessing the VCSEL’s suitability as an illumination source in imaging applications, where spectral diversity is linked to reduced spatial coherence and speckle suppression. The results shown in Fig. 4b reveal that the D-, M-, and P-VCSELs exhibit significantly higher summed intensities than the O- and S-VCSELs, indicating an increase in the number of modes, which correlates with their better imaging performance and reduced speckle, as demonstrated in Fig. 4c, d.

Figure 4c shows images of a USAF (United States Air Force) resolution target illuminated by each VCSEL with the same exposure time. The clarity of these images reveals the spatial coherence of each VCSEL’s emission. Notably, the traditional O-VCSEL and S-VCSEL exhibit a significantly noticeable speckle pattern across the images, which is caused by the high spatial coherence beam^32,33^. While high coherence generally implies sharper images, the coherence-induced speckling actually disrupts image uniformity, introducing unwanted noise. These speckles can be detrimental in imaging systems where smooth, artifact-free illumination is essential. In contrast, the D-, M-, and P-VCSELs deliver much clearer images, with significantly less speckle than the O- and S-VCSELs. Figure 4d further quantifies these observations by providing the speckle images and their corresponding contrast values (*C*) for each VCSEL. The speckle contrast is defined as the standard deviation of the speckle pattern pixels divided by the mean intensity^34^. Higher contrast values in the O-VCSEL (*C* = 0.26) and S-VCSEL (*C* = 0.27) indicate more prominent speckling due to the high spatial coherence. Conversely, the D-VCSEL (*C* = 0.19), M-VCSEL (*C* = 0.17), and P-VCSEL (*C* = 0.15) show progressively clearer images with lower contrast values, confirming their reduced speckle and lower coherence from a large number of incoherent modes, which coincidence with the results shown in Fig. 4b. The supporting coexisting incoherent modes effectively mitigate coherence, leading to smooth images that enhance visual clarity.

Regarding the image and illumination light source, conventional light-emitting diodes (LEDs) have limitations in terms of power^35^. The low brightness and resolution could negatively affect the display quality, making the images less vivid or discernible, especially in well-lit or outdoor environments. Although the engineered random lasers have been investigated to provide low spatial coherence, resulting in speckle-free imaging^36^, most random lasers rely on optical pumping, which impedes their practical use in consumer electronics. Therefore, compact semiconductor low-coherence light sources are ideal for more application scenarios^37^; one of the most popular candidates is the superluminescent diode (SLD), which balances the high power and low speckle between the traditional lasers and the LEDs^38,39^. However, it still suffers from high spatial coherence. Notably, the speckle contrasts achieved by D-, M-, and P-VCSEL are still lower than that reported using the SLD (*C* = 0.2)^40^. More importantly, using the 2D VCSEL arrays, the free-speckle images can be easily achieved by introducing more mutually incoherent VCSELs, which is the main advantage of VCSEL due to its surface-emitting configuration. If the number of light sources is increased to *N*, the speckle contrast will be reduced by a factor of $\frac{1}{\sqrt{N}}$ ^41,42^. Given the highest power efficiency and the lowest speckle provided by the P-VCSELs, fewer devices in the array and much lower energy are needed to achieve speckle-free imaging with the same power. For example, to achieve speckle contrasts below the human perception limit (*C* < 3%)^43,44^, using the data shown in Fig. 4d, an array of 81 S-VCSELs would be needed to achieve that, while only 22 P-VCSELs are needed, which significantly lowers the devices and injection power, as well as the footprint of the VCSEL array. Therefore, given the highest power and the low coherence delivered by P-VCSEL, it is particularly advantageous for imaging systems that require even, high-intensity illumination without artifacts.

**Fig. 5:**
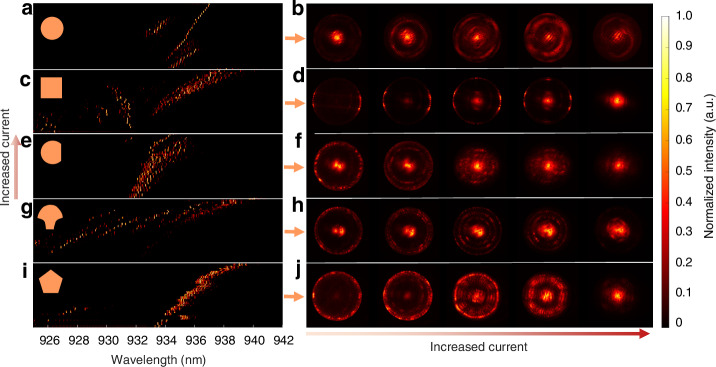
**Spectral and far-field characteristics of VCSELs with different cavity geometries under different current levels**. **a**, **c**, **e**, **g**, **i** Wavelength-resolved spectra for O-, S-, D-, M-, P-VCSEL, respectively, showing the evolution of emission wavelength with increased current. **b**, **d**, **f**, **h**, **j** FFPs at different current levels for O-, S-, D-, M-, P-VCSEL, respectively, demonstrating the evolution of beam profile with increasing drive current for each cavity geometry. Representative cavity shape icons are displayed on the left of each spectra map, with arrows linking to the associated far-fields

We then explore the lasing behavior of VCSELs with these five cavity shapes under varied current injections from around the threshold to the thermal rollover. Figure 5 illustrates how each geometry affects both the spectrum and far-field properties of the VCSELs, revealing the intricate interplay between cavity shape, mode distribution, and beam profile at different current levels. The detailed normalized spectra are provided in Supplementary Fig. S14. These insights are essential for understanding the role of geometry in managing spectral evolution in tailoring VCSELs for specific applications.

For the conventional O-VCSEL, the wavelength-resolved spectra in Fig. 5a show a dominant narrow peak accompanied by a few secondary peaks. The narrow peak may correspond to the mode with the exceptionally high Q-factor shown in previous simulation results. As the current increases, both the main peak and secondary peaks undergo a slight redshift, while the secondary peaks shift from longer to shorter wavelengths at a certain point, likely indicating mode switching. This spectral evolution is mirrored in the FFP shown in Fig. 5b, where the emission pattern begins as a centralized, low-order mode profile. As the current rises, the FFP broadens slightly, suggesting increased mode diversity and a modest decrease in spatial coherence. However, at higher currents, the spectra narrow again, and the FFP reverts to a more centralized pattern, suggesting realignment towards lower-order modes as the VCSEL reaches saturation. This cyclic behavior of spectral width and FFP in the O-VCSEL highlights its ability to maintain a high coherence profile, a desirable characteristic for applications prioritizing high coherence stability.

The S-VCSEL presents unique spectral characteristics, with multiple distinct groups of peaks, as shown in Fig. 5b. Interestingly, as the current increases, the long-wavelength peaks broaden and redshift, whereas the shorter-wavelength peaks exhibit blue shifts before disappearing at high currents. In the FFP, the S-VCSEL starts with a high-frequency outer ring at low currents, corresponding to the short wavelengths (high-frequency) in the spectrum. This observation is well aligned with the previous study on the square VCSEL^45^. As current increases, low-frequency modes emerge in the center (see Fig. 5d), aligning with the appearance of long-wavelength peaks in the spectra. At very high currents, only the centralized low-frequency modes persist, indicating that square geometries stabilize low-frequency modes under high pumping.

The D-VCSEL and M-VCSEL exhibit similar initial lasing behavior, both supporting low- and high-frequency modes even at the threshold. As shown in Fig. 5e, both cavities start with a slightly broad spectral profile at low currents, and the FFPs in Fig. 5f also show that both the bright center and edge rings indicate active lasing modes across a wide frequency range. This phenomenon is suspected because of the curved boundary of the cavity, since other cavities with straight boundaries either start with low-frequency modes or high-frequency modes. However, as the current increases, these two geometries diverge in their spectral response. The D-VCSEL maintains consistent spectral broadening, suggesting controllable multimode lasing and coherence with the current, which is allowed by multimode coexistence within the cavity. This observation is consistent with previous simulations and studies where the D-shaped cavity supports many modes lasing together^8,16^. Correspondingly, the FFP remains relatively diffused without any dominant modes as the current increases, also indicating controllable divergence in the beam profile. This sustained broadening spectrum in the D-VCSEL makes it suitable for applications requiring controlled coherence and beam divergence.

While the M-VCSEL design also supports a combination of low- and high-order modes even at the threshold, it exhibits distinctive lasing modes shaped by its unique geometry, which combines a wide semicircular top section with a narrow stem. These distinct peaks appear and redshift significantly with increased current, as shown in Fig. 5f, unlike the more continuous broadening seen in some other geometries. This indicates selective mode reinforcement within the mushroom shape, where specific modes are favored at higher currents. In the FFP shown in Fig. 5g, these spectral characteristics are mirrored in the appearance and transformation of concentric rings. As the current increases, additional rings appear and intensify, indicating the excitation of higher-order angularly symmetric modes. The evolution of these ring structures in the FFP highlights the M-VCSEL’s ability to maintain a radially distributed beam. At high currents, the FFP shows a diffuse ring pattern with reduced central intensity, suggesting multimode lasing even after saturation without sacrificing overall power, which is consistent with the slowly decreasing power after the rollover shown in Fig. 1f.

Finally, it is worth noting that the P-VCSEL shows the most complex multimode lasing with densely packed peaks at a wide range of currents and intricate and complicated FFPs. Compared to other cavity designs, the P-VCSEL sustains the closely multimode peaks with the least broadening spectrum under the increasing current, as seen in Fig. 5i, which suggests that the pentagonal design supports intense mode competition as they share the same spectrum resource from the gain medium. The FFP in Fig. 5j begins with a ring-shaped emission pattern at low currents, indicating that high-frequency modes lase first. As the current increases, the FFP slowly contracts, correlating with the slight redshifting spectral peaks. Moreover, it is noted that the P-VCSEL’s intricate FFP structure indicates a multi-modal wavefront, making it promising for mode-division multiplexing communication and multi-channel sensing where beam shaping is beneficial. Additionally, the P-VCSEL achieves the highest power among all the geometries, making it suitable for high-power, low-coherence illumination with a broad angular spread, which is ideal for imaging systems requiring speckle-free lighting.

### Polarization dynamics

Unlike edge-emitting lasers, VCSELs normally exhibit unstable polarization behaviors due to their circular cavity and vertical emission, which makes their polarization highly sensitive to internal factors like materials and stress, as well as external factors like temperature and injection current^46,47^. For example, 940-nm VCSELs exhibit polarization behavior affected by the crystal orientation of the semiconductor wafer. The anisotropic properties of the crystal lattice can lead to slight variations in the refractive index along different directions, favoring one polarization axis^48–50^. This effect is particularly evident in devices without significant structural asymmetry, where crystal orientation becomes the dominant factor in determining polarization. However, by incorporating structural modifications such as non-circular cavities, it is possible to influence and stabilize the polarization state, thereby improving the device’s suitability for applications that require a stable polarization output, such as high-speed communication and sensing. It has been demonstrated that the elliptical cavity can stabilize the polarization state of VCSELs, where the dominant polarization direction is along the shorter axis of the elliptical^51^. In continuation of our study on the impact of cavity geometry on the lasing characteristics of VCSELs, here we investigate polarization properties and radio frequency (RF) intensity fluctuation behavior across various cavity shapes.

**Fig. 6:**
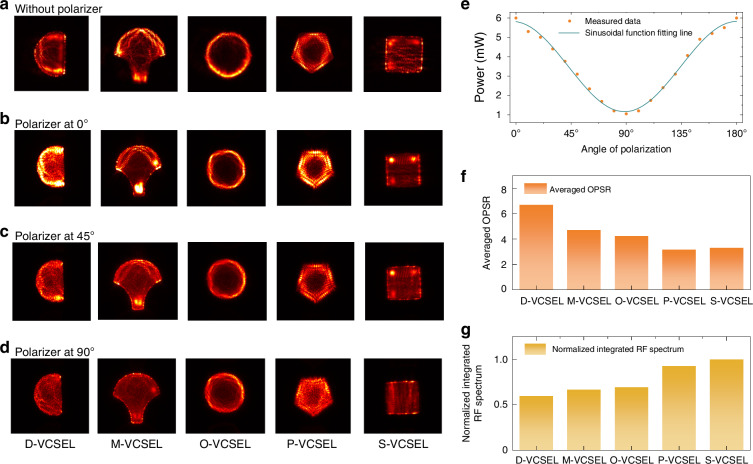
**Polarization-dependent near-field patterns and RF spectrum characteristics of VCSELs with various cavity geometries**. Near-field emission patterns of each VCSEL geometry captured without a polarizer (**a**) and with a polarizer set at angles of 0° (**b**), 45° (**c**), and 90° (**d**). **e** Angular dependence of the optical power, demonstrating the polarization preference of the VCSELs with respect to the crystal orientation of the 940-nm wafer. **f** Averaged orthogonal polarization suppression ratio (OPSR) for each VCSEL geometry, showing the degree of polarization control provided by each design. **g** Normalized integrated RF spectrum for each geometry, calculated from time-domain signals under DC conditions at maximum power, serving as an indicator of signal instability

We measured the NFPs of the VCSELs at different polarization states using a linear polarizer (Thorlabs LPVIS050) placed before the camera. The detailed experimental steps and information are provided in the Supplementary Material. As shown in Fig. 6, it presents the NFPs of each VCSEL geometry with and without polarizers, along with the optical power at different polarization angles, averaged orthogonal polarization suppression ratio (OPSR), and the integrated RF spectrum. This analysis offers key insights into how geometric design affects polarization property and multimode stability, which are essential in numerous photonic applications, including imaging, sensing, and high-speed optical communication. Detailed measurements and results are provided in the supplementary material. Figure 6a–d show the NFPs for each cavity geometry under different polarization conditions, wherein Fig. 6a shows the NFPs without a polarizer, revealing the inherent emission distributions across geometries. When the polarizer is oriented at 0°, which aligns with the [011] direction of the highest output power, the emission profiles reveal the primary polarization modes for each geometry. At this orientation, the emission from each VCSEL geometry becomes markedly more isotropic. This alignment with the direction of maximum power output provides optimal polarization stability and intensity, which is particularly beneficial for applications requiring high output power along a controlled polarization axis. Conversely, when the polarizer is rotated to 90° (aligns with the $[01\bar{1}]$ direction), as shown in Fig. 6d, the power reaches the lowest point, with the emission becoming less concentrated across the whole active region. The previously strong intensity contrast becomes diffused, and the structured patterns seen at 0° polarizer are reduced. This uniform emission at 90° indicates a weakened polarization effect, which suggests that the least preferred modes contribute more evenly across the cavity geometry. The same polarization preference direction was found the same for all devices, with a representation case of the O-VCSEL operated at 50 mA continuous wave (CW) injection shown in Fig. 6e. The power variation along different polarization angles shows the highest power at 0° and the lowest power at 90°, and the changing curve aligns well as a sinusoidal function according to the angle. When the polarizer is rotated to 45°, the NFPs exhibit a combination of two orthogonal modes. Additionally, it is interesting to note that the Fabry–Perot-like modes of the S-VCSEL closely follow the direction of their polarization with distinct orthogonal directions. The experimental results presented here are theoretically predicted with the two-dimensional Maxwell–Bloch equations and have been studied previously^52,53^. The orthogonal polarization emission holds promising potential for practical applications. In particular, the ability to generate multiple polarization channels from a single emitter can enable new functionalities in compact polarization-sensitive sensors, multiplexed optical communication, and photonic computing architectures. For instance, polarization-resolved VCSELs have been demonstrated as sensitive probes for external perturbations such as temperature^54,55^ and mechanical stress^56^. Furthermore, orthogonal polarization states have been used to encode digital information in a polarization-division multiplexing scheme^57^, and to represent binary spin states in Ising-type photonic computing^58^, where they facilitate the solution of combinatorial optimization problems using coupled VCSEL networks. The distinct polarization dynamics also make VCSELs suitable for neuromorphic computing^59^, where fast polarization switching and multistability are used to emulate neuron-like behavior and enable hardware-accelerated optical information processing.

In Fig. 6f, the OPSR further quantifies each geometry’s ability to maintain a stable primary polarization by suppressing orthogonal modes, which is calculated as ${OPSR}=\frac{{I}_{{||}[011]}}{{I}_{\perp [01\bar{1}]}}$, where ${I}_{{||}[011]}$ is the measured highest optical intensity of the dominant polarization direction long [011], and ${I}_{\perp [01\bar{1}]}$ is the lowest intensity of the orthogonal polarization along the lattice direction $[01\bar{1}]$. The high OPSR values observed for the D- and M-VCSELs reflect their ability to stabilize emission along the crystal-preferred polarization axis, effectively reducing leakage into the orthogonal state. Notably, both of these cavity designs feature a combination of straight and curved boundaries, and the two configurations start with multimode lasing around the threshold. This result somehow indicates that irregular geometries can leverage the intrinsic anisotropy of the 940-nm wafer to enhance polarization stability by designing the alignment between the crystal axis and the straight boundary of the aperture.

Furthermore, we calculated the RF power spectrum of each VCSEL by performing a Fourier transform on the time-domain intensity fluctuations, recorded under DC operation at the highest output power without polarization selection. As shown in Fig. 6g, we present the integrated RF spectra from 0 to 10 GHz, limited by the bandwidth of the photodetector. The results are normalized to the value of the S-VCSEL, which exhibits the highest integrated RF power. This metric acts as an indicator of emission stability^14^: lower integrated RF power corresponds to reduced fluctuation levels, indicating higher temporal stability in the optical emission. Consistent with their high OPSR values, the D-VCSEL and M-VCSEL exhibit the lowest integrated RF powers, suggesting more stable emission behavior. This stability can be attributed to the strong polarization preference along the crystal axis, which suppresses polarization switching, reduces mode competition, and stabilizes temporal dynamics. In contrast, the P-VCSEL and S-VCSEL display the highest integrated RF values, consistent with their low polarization preference (i.e., low OPSR), which permits strong coupling and interaction between multiple modes and polarization states. This lack of polarization constraint increases the likelihood of dynamic switching and beating between modes, resulting in a high-frequency-rich RF spectra indicative of chaotic or turbulent multimode lasing dynamics. While such dynamics are typically undesirable in conventional laser applications where coherence and stability are prioritized, they are advantageous for emerging applications that demand high entropy and complex temporal behavior. These include ultrafast physical random number generation^14,60^ and photonic reservoir computing^26,27^, where nonlinear modal coupling and broadband temporal fluctuations can be exploited as a source of computational richness and unpredictability^14,27^.

## Discussion

This study explores the impact of cavity geometry on the optical properties of broad-area VCSELs, revealing that non-circular geometries offer distinct advantages in managing gain utilization, enhancing power output, beam shaping, and tuning spatial coherence. Our results highlight the fundamental role of asymmetric cavities in affecting multimode behavior and overall device performance. Specifically, breaking the transverse symmetry of a VCSEL cavity leads to profound changes in its lasing behavior. These changes originate from a redefinition of the boundary conditions that govern the formation and selection of optical eigenmodes within the cavity. In conventional circular VCSELs, the radial symmetry supports WGMs with well-defined spatial profiles. However, when the cavity adopts asymmetric shapes, such as D-shaped, mushroom-shaped, or pentagonal geometries, the modal landscape becomes spatially distorted. This symmetry-breaking fundamentally alters the modal competition dynamics and reshapes the field distributions within the cavity. Several key performance characteristics are affected. First, the spatial overlap between lasing modes and the gain region is changed, which impacts gain utilization, slope efficiency, and overall output power. Second, interference between asymmetric transverse modes leads to complex far-field beam profiles and reduced spatial coherence. Third, the cavity boundary also influences polarization behavior, inducing instabilities or reorientation of the polarization state during multimode operation.

The key characteristics of each cavity with their corresponding potential applications are concluded in the Table 1. These findings provide critical insights into advancing VCSEL designs, suggesting new pathways for shaping VCSELs tailored to specific application requirements. While a complete theoretical treatment is beyond the scope of this experimental work, the results establish a clear physical rationale for the geometry-induced effects observed, which forms a foundational framework for understanding how cavity design impacts emission characteristics in multimode VCSELs, providing a practical and insightful guide for future simulation and theory-driven studies.

**Table 1:** Summary of cavity-dependent characteristics and their potential applications

Cavity Geometry	Key Characteristics	Potential Applications
O-VCSEL (Circular)	∙ Lowest threshold current ∙ Few-mode lasing (WGMs) ∙ Strong mode suppression	❖ Coherent communication ❖ Precision sensing
S-VCSEL (Square)	∙ Orthogonal polarization modes ∙ Fabry–Perot-like mode profiles	❖ Polarization-resolved sensing ❖ Polarization-division multiplexing
D-VCSEL (D-shaped)	∙ Uniform Q-distribution ∙ Controllable multimode lasing ∙ High polarization stability	❖ Speckle-free illumination ❖ Low-coherence imaging ❖ Free-space projection
M-VCSEL (Mushroom)	∙ Concentric angular modes ∙ Intermediate spatial coherence	❖ Structured-light generation ❖ Optical coherence tomography
P-VCSEL (Pentagon)	∙ Highest optical power ∙ Rich multimode dynamics ∙ Strong mode interaction	❖ Entropy source ❖ Photonic reservoir computing ❖ Random number generation

One of the most significant findings is that the pentagonal VCSELs exhibited double the optical power compared to traditional circular VCSELs. This power enhancement is attributed to the cavity’s ability to distribute gain more effectively across the aperture, thereby improving overall energy utilization. However, this design also introduces increased polarization instabilities, leading to more complex multimode dynamics and polarization interaction, especially at higher injection currents, which can be used in applications requiring fast and complex dynamics, like entropy sources for signal encoding and encryption. In contrast, the D- and mushroom-shaped geometry demonstrated both high power output and superior mode stability, making it ideal for applications requiring multimode lasing stability, such as structured illumination and imaging systems. While less effective at maximizing power output, the square geometry provided distinct orthogonal polarization states, facilitating enhanced polarization control. These findings highlight the potential of non-circular geometries to offer more tailored properties in various applications.

While this study provides a characterization and analysis of cavity geometry effects on VCSEL performance, future research could explore several directions to further enhance our understanding of the chaotic-cavity VCSELs. For instance, while the simulations in this study are performed under passive (cold-cavity) conditions, these results do not account for active lasing effects such as gain saturation, spatial hole burning, carrier heating, and thermal lensing due to the complexity and computational resources. These factors can impact threshold behavior, modal competition, and spectral dynamics under real-world operation. To model these effects more comprehensively, future work will focus on multi-physics simulations under active lasing conditions. Approaches such as the Steady-State Ab Initio Laser Theory (SALT) offer promising frameworks for simulating lasing in complex, non-Hermitian gain systems^61,62^, and can be extended to include broadband, multimode VCSELs. Although beyond the scope of this study, such advanced simulation efforts are an integral part of our ongoing research and will be key to optimizing future VCSEL architectures for tailored coherence, stability, and entropy-rich dynamics. Additionally, experimental studies that map the spatial distribution of carriers and correlate it with optical characteristics could provide insights into optimizing current injection for each cavity shape. Additionally, hybrid cavity designs combining features of multiple geometries could uncover new performance advantages by integrating high power output with stable polarization and low coherence from different devices.

In conclusion, this study demonstrates the importance of cavity geometry in shaping the optical, spectral, coherence, beam profile, and polarization characteristics of VCSELs. By systematically analyzing five distinct geometries, we prove that non-circular cavities can significantly enhance gain utilization, increase power output, and manage beam and multimode lasing behavior more effectively than traditional circular VCSELs. Our findings contribute to designing and applying VCSELs across diverse fields, from secure communications and sensing to imaging and illumination. This work lays a foundation for future explorations of VCSEL design, paving the way for optimized devices that meet the growing demands of modern optoelectronic systems.

## Materials and methods

### VCSEL wafer and fabrication

VCSELs with varying geometries were fabricated using a commercial 940-nm VCSEL wafer. The epi-layers are grown on an *n*-GaAs substrate (100) and comprise an *n*-type GaAs buffer layer, an *n*-type AlGaAs distributed Bragg reflector (DBR) stack (38 pairs) as the bottom mirror, a *λ*/2-thick cavity with five quantum wells designed to emit around 940 nm, a stack of 20-pair *p*-type AlGaAs DBR layers as the top reflector (including a layer with an Al composition of 98% to form the oxide aperture), and a 20-nm *p*++-GaAs layer. During the fabrication, initially, mesas were formed using inductively coupled plasma reactive-ion etching (ICP-RIE) with a silicon oxide mask, reaching a depth of approximately 3850 nm. Subsequently, the oxidation process was performed for apertures crucial for both current and optical confinement. After oxidation, the hard mask was removed, followed by a silicon oxide sidewall insulation layer deposition using plasma-enhanced chemical vapor deposition (PECVD). This layer was then patterned through photolithography and reactive-ion etching (RIE). The subsequent steps involved the patterning of both negative and positive metal pads using lift-off. An annealing step was finally implemented. The details can be found in the previous work^14^.

### VCSEL characterization

The optical power of the VCSELs is measured using a Keithley 2520 laser testing system with a thermoelectric controller (TEC) set at 15 °C. Power measurements were conducted using a calibrated silicon-based detector (SCC-PMSI) situated on a Labsphere integrating sphere. The spectra measurement of the VCSELs utilizing the Keithley 2510 system with a TEC (LDC-3900 Modular 4-Channel Laser Diode Controller) set at 17 °C. The wavelengths are measured by the Hyperfine spectrometer (LightMachinery) with a resolution of 1 pm.

### VCSEL simulation

A 3D simulation model for a circular cavity is established with a cavity diameter of 5 μm due to the limited computation resources. Other cavities are designed to have a similar active region as the circular cavity. The center frequency is chosen as 320 THz to monitor the emission wavelength around 940 nm. The height of the 3D cavity is calculated as $\frac{{\rm{\lambda }}}{2{\rm{n}}}$, where λ is the vacuum wavelength, and n is the refractive index of GaAs, which is set as 3.5 to simplify calculations. To model the high reflectivity of the top and bottom distributed Bragg reflectors (DBRs), perfect electric conductors (PECs) are applied, ensuring total reflection along the z-axis. The cavity is surrounded by an air layer to simulate the sidewall reflections and associated losses. Additionally, a perfectly matched layer (PML) is placed outside the air region to absorb any light reaching the boundaries, preventing artificial reflections. For the 2D circular cavity simulation, wave vectors in the z-direction were neglected, with no boundary condition applied vertically. The details of the simulation and results can be found in the supplementary material.

## Supplementary information

Supplementary material-Shaping the Light of VCSELs through Cavity Geometry Design

## Data Availability

The data that support the findings of this study are available from the corresponding author upon reasonable request.
