# Supplementary material for: Shaping the light of VCSELs through cavity geometry design
Source: Light Sci Appl. 2025 Sep 28;14:344. doi: 10.1038/s41377-025-01996-7 (PMC12477291; doi:10.1038/s41377-025-01996-7)
Supplement: Supplementary file 1 — Supplementary material-Shaping the Light of VCSELs through Cavity Geometry Design [file 41377_2025_1996_MOESM1_ESM.pdf]

Supplementary material

Shaping the Light of VCSELs through Cavity Geometry Design

Hang Lu,[a)] Omar Alkhazragi,[a)] Heming Lin, Tien Khee Ng, and Boon S. Ooi[*]

Photonics Laboratory, Electrical and Computer Engineering Program, Division of Computer, Electrical, and Mathematical Sciences and Engineering (CEMSE), King Abdullah University of Science and Technology (KAUST), Thuwal, 23955-6900, Kingdom of Saudi Arabia.

[a)] H. Lu and O. Alkhazragi contributed equally to this work.

[*] Corresponding author. Electronic mail: boon.ooi@kaust.edu.sa.

Section 1: Power characteristics

To illustrate the impact of cavity geometry on the lasing performance, we first measured the light–current (L–I) characteristics of VCSELs with five distinct aperture shapes. Fig. S1 shows representative L–I curves for each geometry, where the device dimensions are annotated in the insets. These devices were chosen from the same cell to ensure consistent fabrication conditions. As observed, the optical threshold current, slope efficiency, and peak output power vary significantly with geometry. Notably, the pentagonal VCSEL achieves the highest output power (~24.5 mW), while the circular geometry yields the lowest (~14 mW). These representative curves provide a first look at the static performance differences introduced by transverse cavity design and serve as a baseline for further statistical and dynamic analysis.

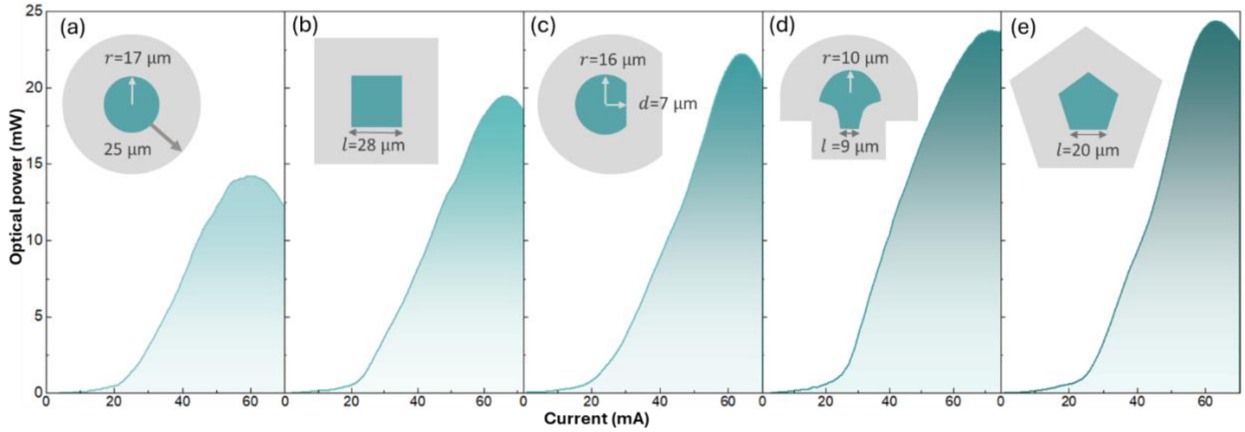

Fig. S1. Light–current characteristics of VCSELs with different cavity geometries. Representative L–I curves of five VCSEL geometries: (a) circular (O-VCSEL), (b) square (S-VCSEL), (c) D-shaped (D-VCSEL), (d) mushroom-shaped (M-VCSEL), and (e) pentagonal (P-VCSEL).

Section 2: Far-field and Near-field measurement

To characterize the near-field (NFP) and far-field (FFP) patterns of the vertical-cavity surface-emitting laser (VCSEL), we employed the optical setup illustrated in Fig. S2. This system allows the simultaneous acquisition of near-field and far-field measurements, ensuring precision and consistency in data collection. The VCSEL under test was mounted on a thermoelectric cooler (TEC) set at 17°C to maintain thermal stability during the experiment, mitigating thermal-induced performance variations. The emitted light was collected using a 50× objective lens (0.45 NA) and then directed to a dichroic mirror (Thorlabs, DMLP650 - Ø1) that splits the beam into two distinct optical paths for near-

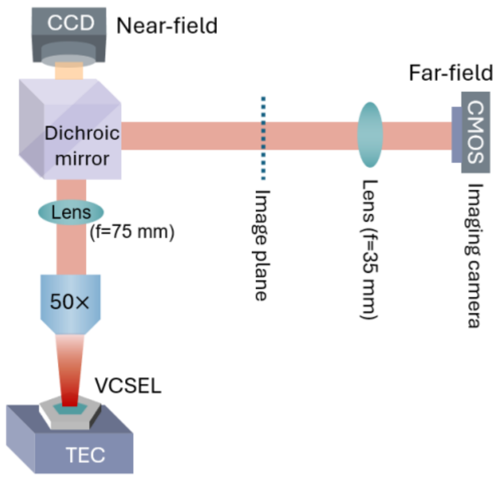

Fig. S2: Experimental setup for near-field (NFP) and far-field (FFP) pattern measurements of the VCSEL. The VCSEL light is collected by a 50× objective lens and split into two paths by a dichroic mirror. The vertical path directs the beam to a CCD camera for near-field imaging, while the horizontal path uses a lens f=35mm to project the far-field pattern onto a CMOS camera using a 4f system.

field and far-field imaging. The far-field patterns were projected onto a CMOS camera (Thorlabs, CS165MU1) using a 35 mm focal length lens, which, together with a 75 mm vertical pathway lens, forms a 4f system to project the far field [42–44]. Near-field imaging was conducted using a CCD camera (Thorlabs, DCU224C), capturing intricate details of the emitted patterns.

A series of characterization tests were conducted using VCSELs with various cavity geometries, including circular (O-VCSEL), square (S-VCSEL), D-shaped (D-VCSEL), mushroom-shaped (M-VCSEL), and pentagon shaped (P-VCSEL) cavities. Representative results are presented in Figs. S3-7. The NFPs captured by the CCD camera are displayed in the top row. These images reveal the spatial intensity distribution at the VCSEL cavity. The Fourier transform (FFT) of the near-field patterns, shown in the middle row, was calculated using MATLAB, providing a representation of the spatial frequency domain. The bottom row displays the FFPs, obtained by the CMOS imaging camera through the 4f optical system. These far-field images represent the angular distribution of the emitted light, providing critical information about the beam quality, divergence, and coherence. The strong agreement between the near-field FFT and the far-field patterns confirms the optical system's effectiveness in capturing the Fourier relationship between the NF and FF. These results collectively demonstrate the strong influence of cavity geometry on the optical properties of VCSELs. The NFPs and FFPs showcase the dynamic evolution of spatial coherence, mode competition, and angular emission characteristics with increasing current levels (from left to right), which highlights the role of cavity design in tailoring VCSEL performance for specific applications.

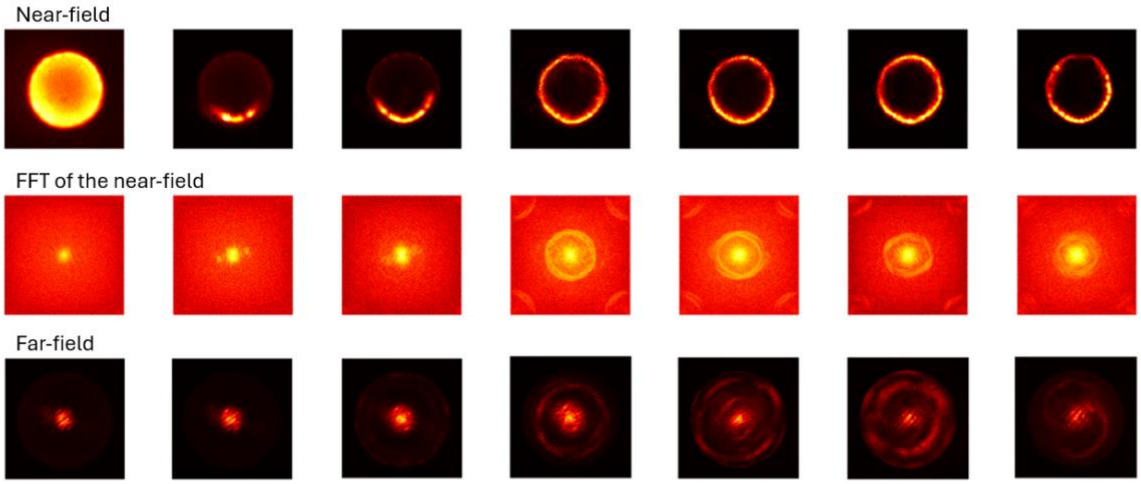

Fig. S3: NFPs, FFT of the NFP, and FFPs of the O-VCSEL at increasing current levels from left to right.

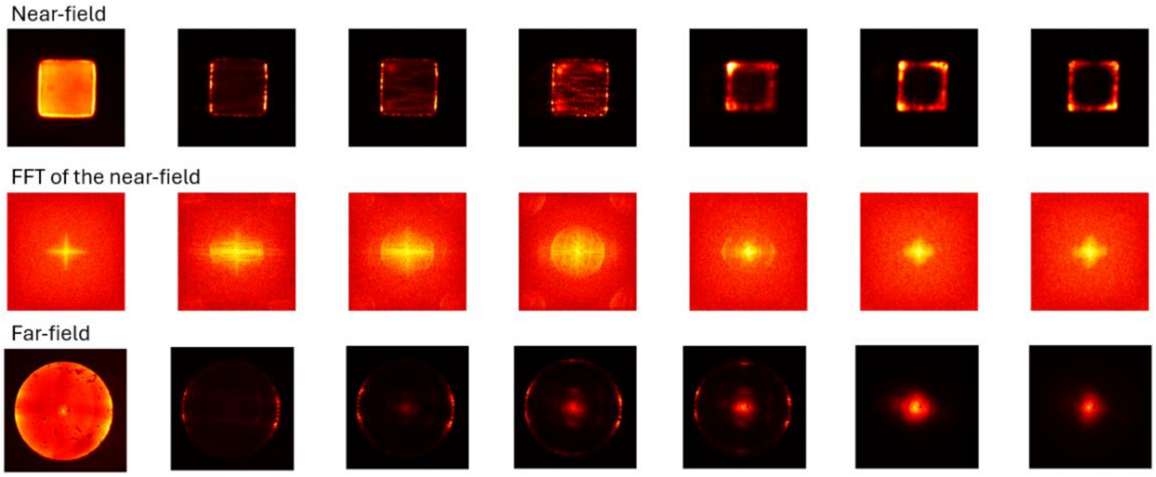

Fig. S4: NFPs, FFT of the NFP, and FFPs of the S-VCSEL at increasing current levels from left to right.

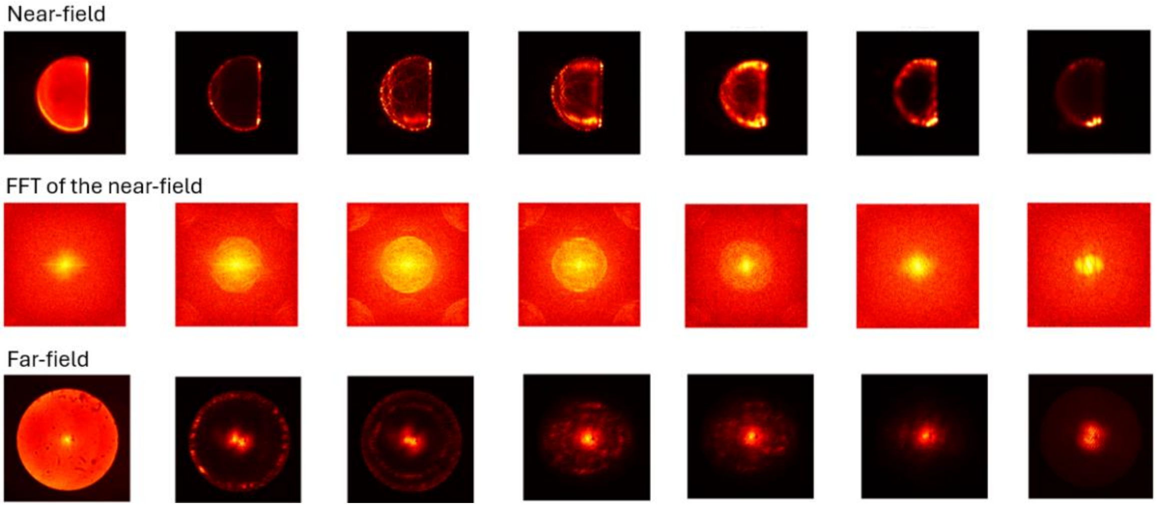

Fig. S5: NFPs, FFT of the NFP, and FFPs of the D-VCSEL at increasing current levels from left to right.

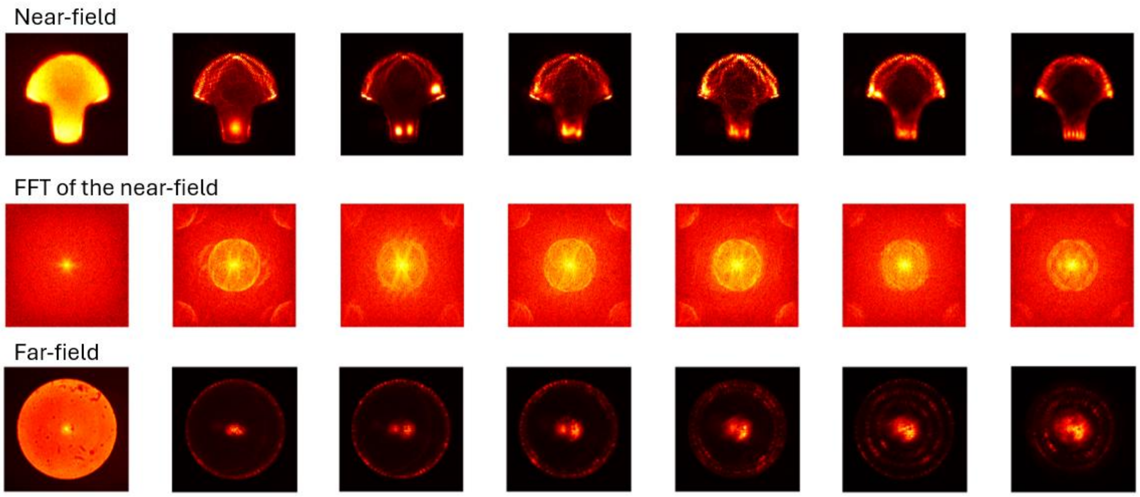

Fig. S6: NFPs, FFT of the NFP, and FFPs of the M-VCSEL at increasing current levels from left to right.

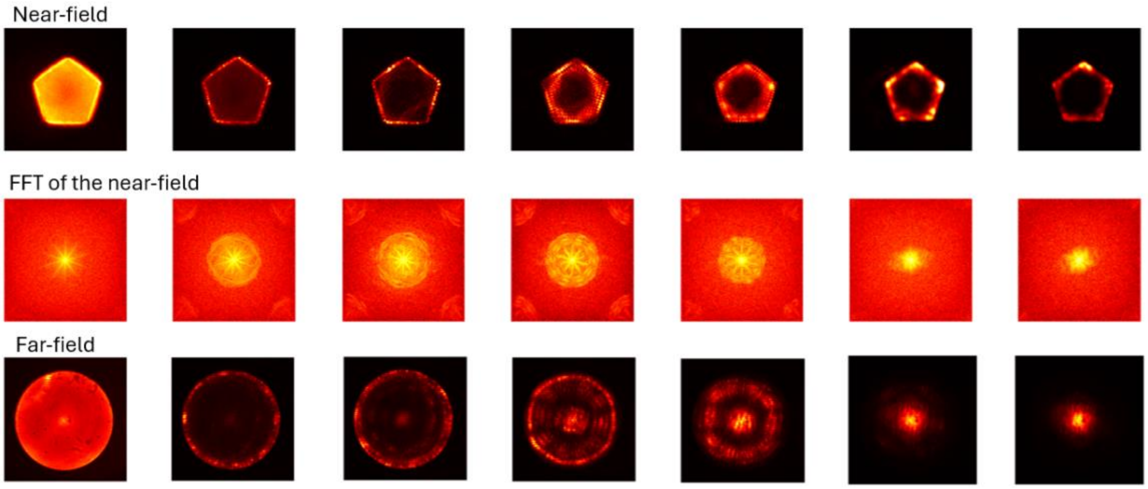

Fig. S7: NFPs, FFT of the NFP, and FFPs of the P-VCSEL at increasing current levels from left to right.

Section 3: Spectra measurement results

To further observe the spectral behavior of VCSELs with different cavity geometries, we present a set of normalized spectra recorded under increasing injection currents. These normalized spectra are scaled to their peak intensity at each current level. This normalization emphasizes relative modal contributions and reveals how the distribution and evolution of lasing modes vary across geometries, independent of output power. In the experiment, each VCSEL was mounted on a temperature-stabilized thermoelectric cooler (TEC) platform stabilizing at 17 ℃ and driven by a Keithley 2520 current source. Spectra were collected at multiple injection current levels using a high-

resolution hyperfine spectrometer (LightMachinery) with a resolution of 1 pm, spanning the sub-threshold to saturate regimes, for all five cavity geometries.

Fig. S8 summarizes the normalized spectral evolution, where Fig. S8(a) shows wavelength-resolved spectral maps for all geometries, stacked from bottom to top by increasing injection current. Fig. S8(b) to S8(f) display the individual normalized spectra for the O-, S-, D-, M-, and P-VCSELs, respectively. Each geometry exhibits distinct spectral behaviors. For example, the O-VCSEL (Fig. S8(b)) demonstrates a dominant narrow spectral peak at low injection current, accompanied by a few weaker side modes. These dominant modes likely correspond to high-Q whispering gallery modes that reach threshold early and suppress others due to gain competition. As current increases, spectral broadening occurs with new modes emerging, but the emission eventually reconverges toward low order as gain saturates. The S-VCSEL (Fig. S8(c)) exhibits a more complex mode evolution. The normalized spectra contain multiple distinct peaks even near threshold, distributed across both short and long wavelengths. As the current increases, longer-wavelength peaks redshift and disappear, while high-frequency peaks persist. The D-VCSEL (Fig. S8(d)) displays a broad and relatively uniform spectral profile, with multiple peaks spanning a wider wavelength with increasing current. The M-VCSEL (Fig. S8(e)) exhibits distinctive spectral peaks that shift with current. These peaks, separated by large gaps, suggest geometry-induced selection and amplification of specific modes. The P-VCSEL (Fig. S8(f)) supports densely packed, closely spaced peaks, suggesting intense interaction among many simultaneously lasing modes. These peaks remain relatively stationary across current levels, indicating strong spectral stability without large shifts.

These results collectively confirm that cavity geometry fundamentally shapes the spectral and spatial evolution of multimode lasing in VCSELs. The normalized spectral profiles provide insight into mode competition and threshold dynamics, while the corresponding FFPs illustrate how geometry dictates the spatial coherence and modal diversity of the emitted beam. This understanding is critical for engineering VCSELs tailored to specific application needs, whether for coherent communication, entropy generation, or low-coherence high-power lighting.

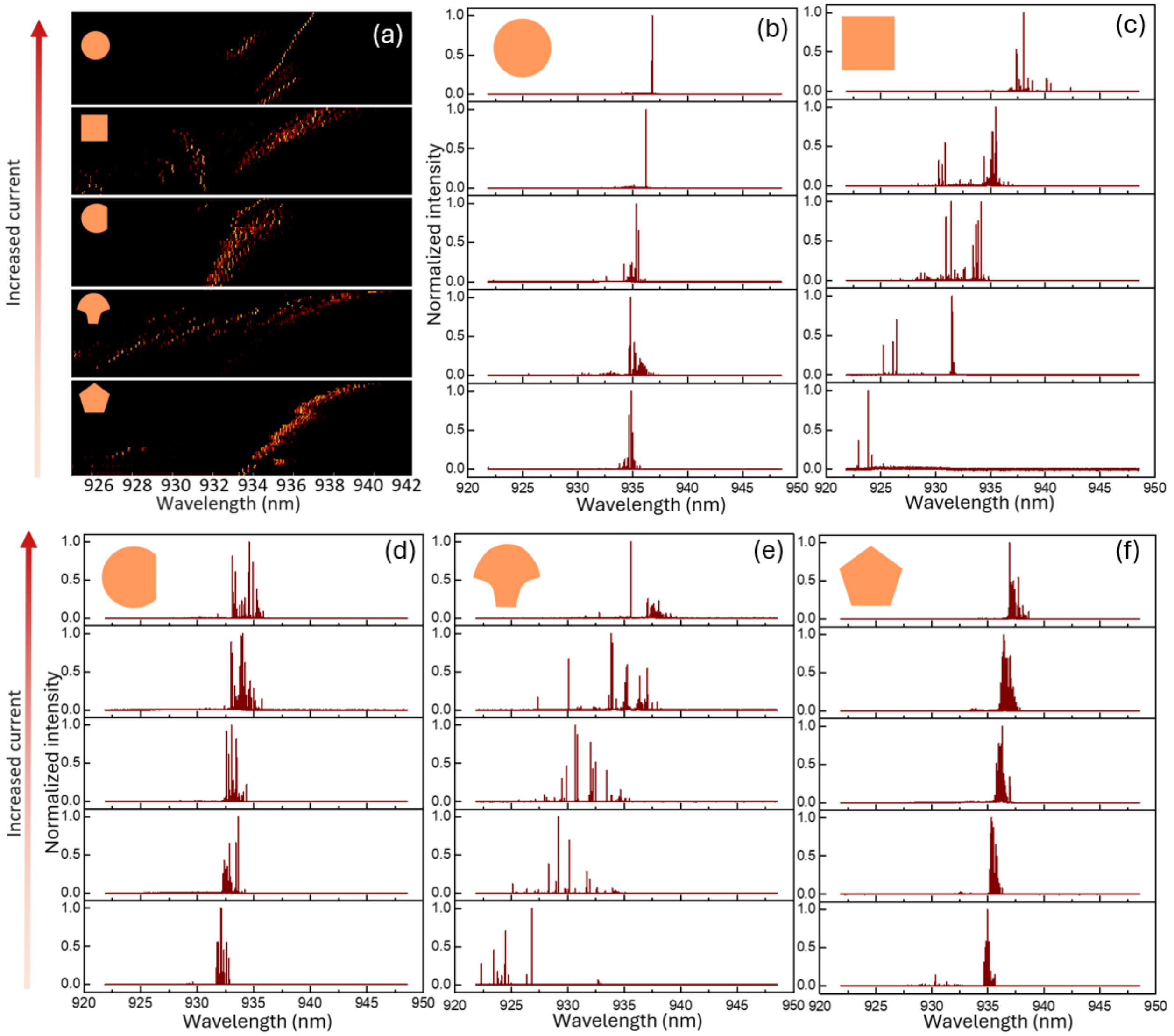

Fig. S8. Normalized spectral evolution of VCSELs with different cavity geometries as a function of injection current. (a) Normalized wavelength-resolved spectra for all five VCSEL geometries under increasing current, where each spectrum is scaled to its peak intensity at each injection level. (b–f) Corresponding normalized spectra for individual geometries: (b) O-VCSEL, (c) S-VCSEL, (d) D-VCSEL, (e) M-VCSEL, and (f) P-VCSEL. For each geometry, multiple spectra are shown at increasing injection levels from bottom to top.

Section 4: Polarization measurement results

To analyze the impact of polarization on the NFP and FFP patterns of the VCSELs, we used the modified experimental setup illustrated in Fig. S9. A linear polarizer (Thorlabs LPVIS050) was introduced into the optical path to selectively analyze the emission characteristics at different polarization states. The polarizer was placed before the imaging cameras in both the NFP and FFP measurement paths. In Fig. S9(a), the setup for NFP measurements is shown. A linear polarizer was positioned between the dichroic mirror and the CMOS camera in the near-field path. The CCD

camera was used for additional probing of the system. By rotating the polarizer, we were able to capture the spatial intensity distributions of the VCSEL emission at specific polarization angles. For the FFP measurements, Fig. S9(b) illustrates the setup. The polarizer was placed in front of the CMOS camera at the end of the far-field path. This allowed the angular intensity distribution to be filtered based on polarization. The far-field images recorded at varying polarization angles highlight the evolution of beam divergence, coherence, and angular distribution as influenced by the VCSEL cavity geometry and polarization state.

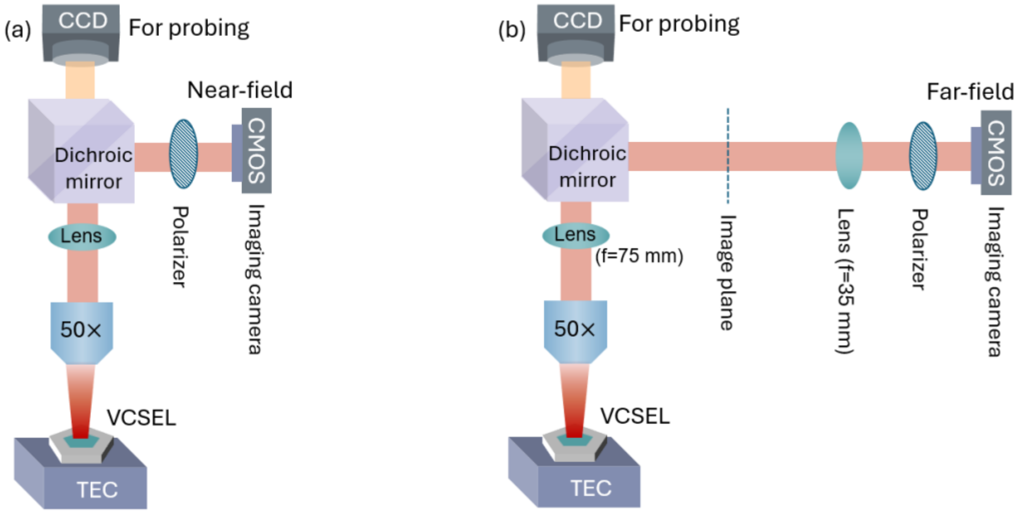

Fig. S9: Experimental setup for NFP and FFP measurements with polarization analysis. (a) Setup for NFP measurements, where a linear polarizer (Thorlabs LPVIS050) is placed in front of the CMOS camera to capture polarization-dependent near-field patterns. (b) Setup for FFP measurements, where the polarizer is positioned before the CMOS camera to analyze the polarization-dependent angular intensity distribution.

The results shown in Figs. S10–14 highlight the distinct influence of cavity geometry on polarization sensitivity and emission dynamics. For the O-VCSEL (see Fig. S10), the NFP and FFP patterns exhibit minimal changes across polarization angles. The near-field remains highly uniform, and the far-field retains its circular symmetry. The 0° polarization state corresponds to the highest emitted power, with fewer variations in intensity and angular distribution observed under other polarization states. This behavior reflects the inherent isotropy of the circular cavity. In contrast, the S-VCSEL shown in Fig. S11 demonstrates significant polarization-dependent variations. The NFPs alternate between horizontal and vertical modal structures as the polarization angle changes, highlighting the dominance of orthogonal polarization modes. The FFPs display corresponding intensity variations, with pronounced alignment along the square cavity's edges. These results imply the strong coupling between the cavity geometry and polarization modes, which plays a critical role in defining the emission characteristics.

The D-VCSEL illustrated in Fig. S12 reveals pronounced asymmetry in its polarization-dependent emission. As indicated by the large divergence angle shown in the FFP at 0 degrees, it is noticed that high-frequency modes are mainly localized along the curved edge of the cavity in the near-field, which is similar to the M-VCSEL shown in Fig. S13, which exhibits highly localized high-frequency modes at the curved edge of the cavity.

For the P-VCSEL shown in Fig. S14, the NFPs reveal FP-like spatial modes along each straight boundary of the pentagon, especially at 0°, where these modes dominate due to alignment with the highest emitted power. The FFPs also show pronounced features at the large divergence angle aligned with these dominant high-frequency modes. At 90°, however, the spatial modes are distributed approximately across the entire cavity, with no pronounced dominance of specific boundaries. During the experiment, it was noticed that the modes are strongly hopping and changing over time, which is consistent with our observation that P-VCSEL exhibits ultrafast dynamics.

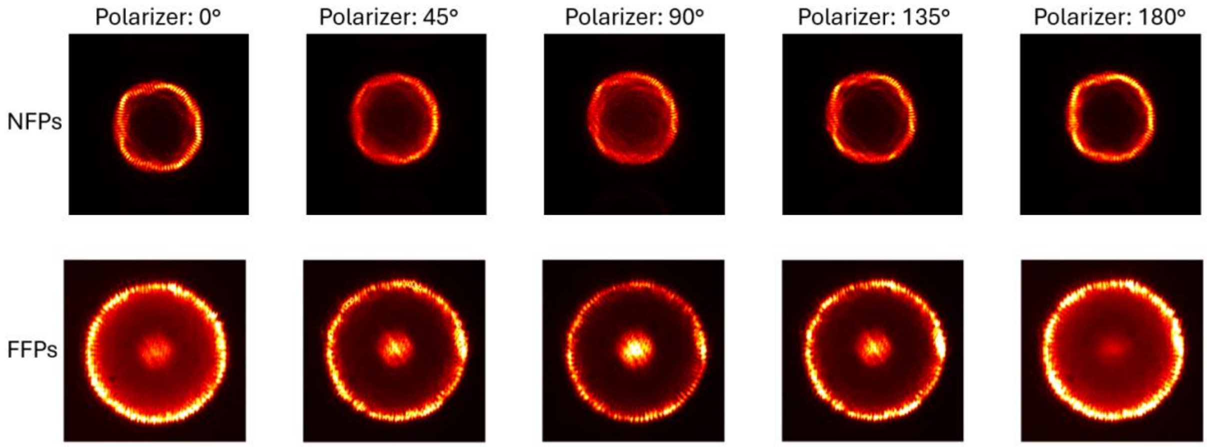

Fig. S10: Polarization-dependent NFPs and FFPs of the O-VCSEL.

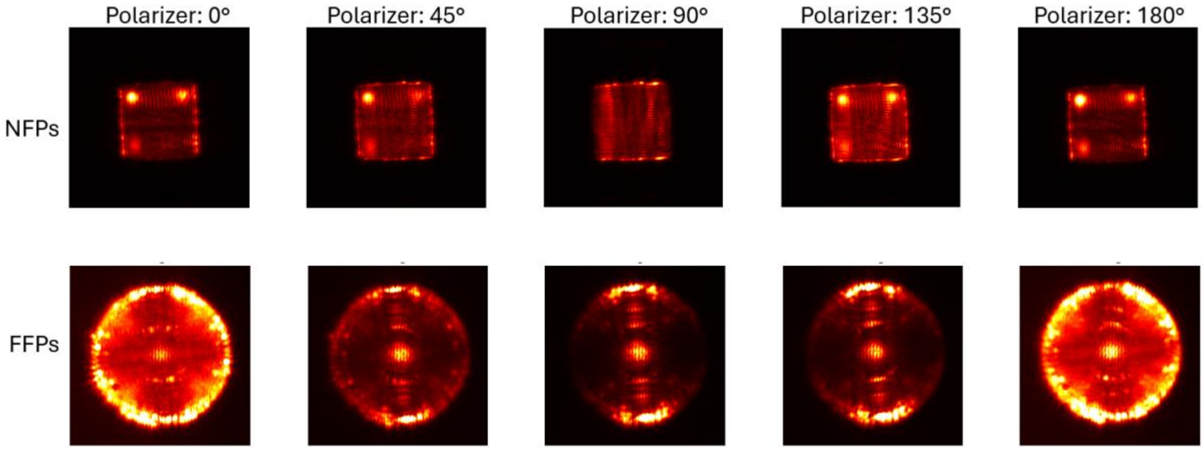

Fig. S11: Polarization-dependent NFPs and FFPs of the S-VCSEL.

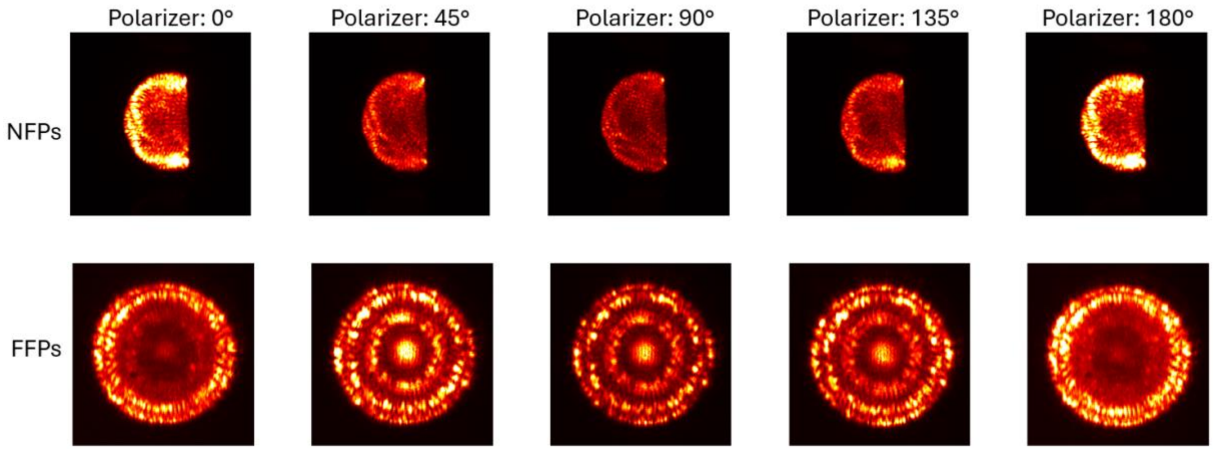

Fig. S12: Polarization-dependent NFPs and FFPs of the D-VCSEL.

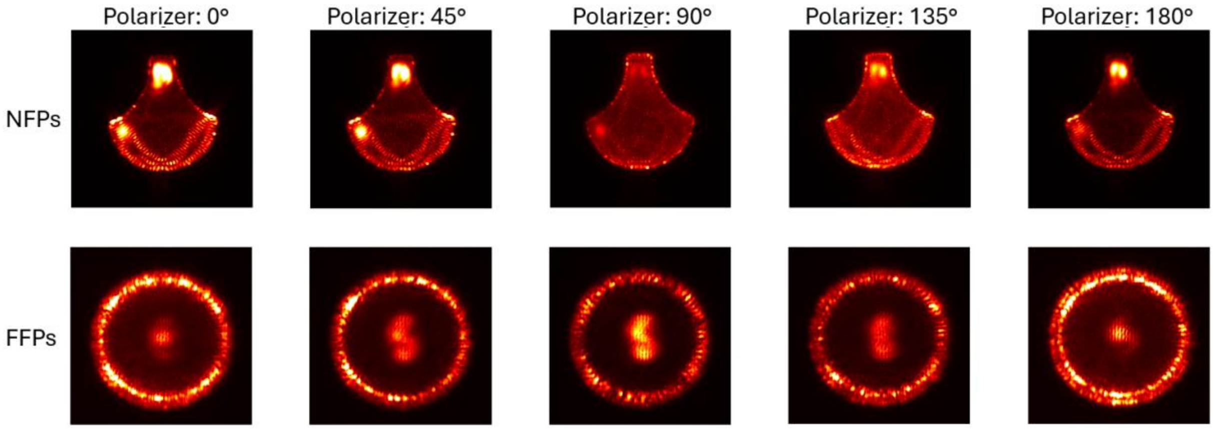

Fig. S13: Polarization-dependent NFPs and FFPs of the M-VCSEL.

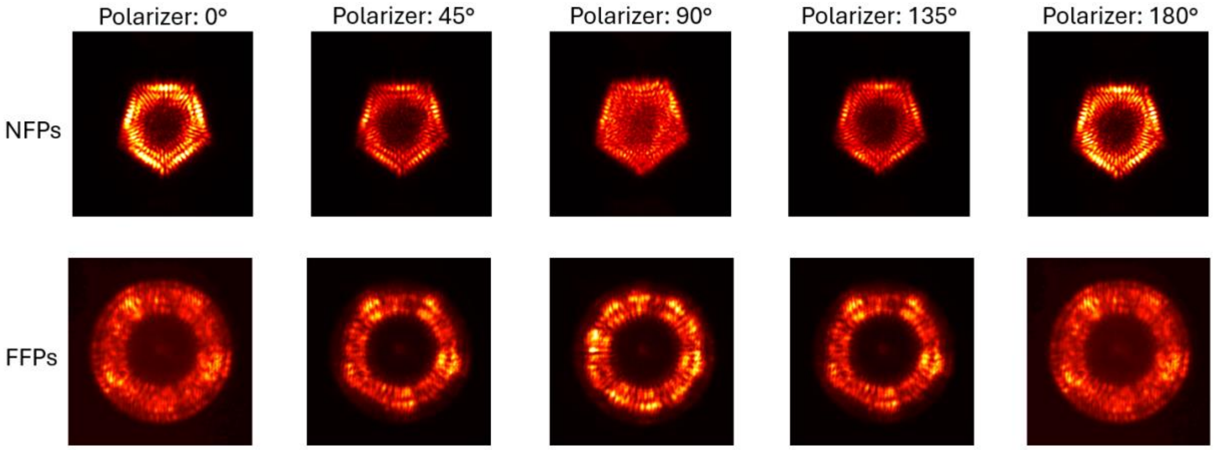

Fig. S14: Polarization-dependent NFPs and FFPs of the P-VCSEL.

Section 5: Simulation results

To evaluate the quality (Q) factors of various VCSEL cavity geometries, a comprehensive 3D simulation model was constructed using COMSOL Multiphysics. A circular cavity with a diameter of 5μm was chosen as the baseline model to optimize computational efficiency. Other cavity geometries, including square, D-shaped, mushroom-shaped, and pentagon-shaped designs, were constructed to maintain comparable active region sizes to the circular cavity. The height of each cavity was determined using $d=\lambda/2n$, where $\lambda=940$ nm, represents the emission wavelength, and $n=3.5$ is the refractive index of GaAs. This simplification ensured consistency across all cavity types while reducing computational complexity. High reflectivity at the top and bottom surfaces, mimicking distributed Bragg reflectors (DBRs), was modeled using perfect electric conductor (PEC) boundary conditions. This assumption ensures total reflection along the z-axis, effectively confining the light vertically within the cavity. To simulate the surrounding environment and account for sidewall losses, each cavity was enclosed by an air region. Additionally, a perfectly matched layer (PML) was applied at the outer boundaries of the simulation domain. The PML absorbed any light reaching the edges, eliminating artificial reflections and providing an open-space boundary condition. The simulations were conducted at a center frequency of 320 THz to monitor the cavity modes near the 940 nm wavelength. Extremely fine meshing was employed to accurately resolve the electric field distributions, particularly near the cavity edges and interfaces. The electromagnetic wave frequency domain module was used to calculate the electric field (E) and determine the Q-factors for each cavity. The Q-factor was computed as the ratio of the stored energy to the energy lost per cycle, incorporating contributions from radiative losses, sidewall scattering, and material absorption.

For validation and computational efficiency, 2D simulations were also performed for the circular cavity. In the 2D models, wave vectors in the z-direction were neglected, and no boundary conditions were applied vertically. These simulations provided insights into the lateral mode behavior while significantly reducing computational load. The comparison between 3D and 2D simulations reveals consistent trends in the Q-factors of the VCSEL cavity modes, as shown in Fig. S15. The top 100 modes with the highest Q-factors are ranked, and their normalized values are presented for both simulation approaches. The results demonstrate strong agreement between the 3D and 2D models, validating the computational simplifications applied in the 2D simulation. Slight deviations observed in lower-ranked modes may be attributed to the omission of vertical wave vector components in the 2D simulation, affecting certain modes' detailed confinement. The accompanying experimental result for a fabricated O-VCSEL with a diameter of 34 μm shows a consistent distribution of high-Q modes compared to the simulated spatial mode results of a smaller

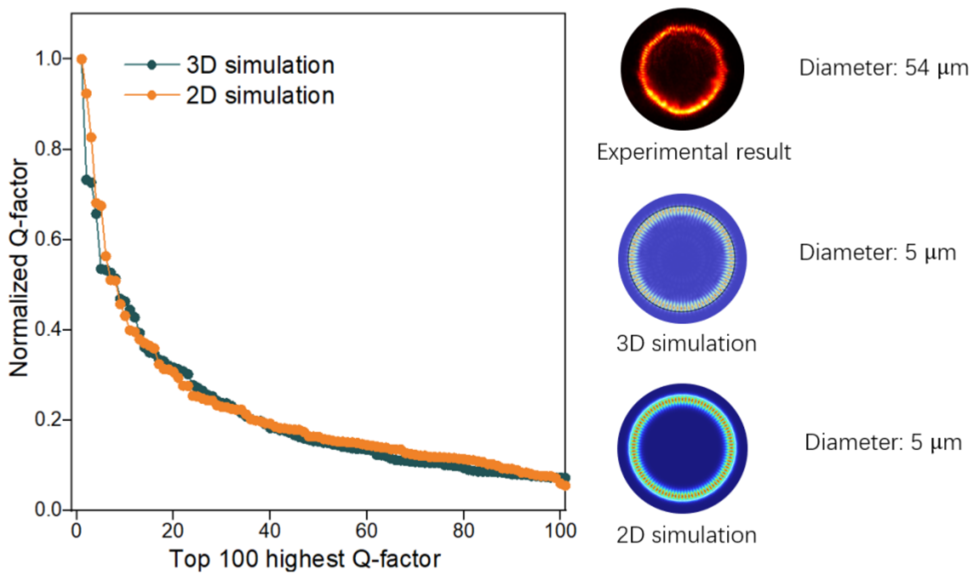

Fig. S15: Comparison of normalized Q-factors for the top 100 modes between 3D and 2D simulations of a circular VCSEL cavity with a diameter of 5 µm.

circular cavity (diameter: 5 µm). This confirms that the mode confinement and optical losses are well-represented in the simulations. The electric field intensity distributions from both 3D and 2D simulations further validate the mode profiles, exhibiting similar spatial patterns and high-Q confinement along the cavity boundaries.

In the main manuscript, the Q-factor distributions of different VCSEL cavity geometries were obtained via 3D eigenmode simulations conducted under passive (cold-cavity) conditions. Due to computational resource limitations, the simulated aperture size was set smaller than that of the actual fabricated device. To evaluate the scalability of the simulation results, we conducted an additional simulation using a larger aperture size. In this extended simulation, the diameter of the O-VCSEL was increased from 5 µm to 20 µm, which is closer to the experimental device diameter (~34 µm). For consistency, the P-, D-, and M-VCSEL geometries were also scaled up to match the area of the larger O-VCSEL. Because 3D simulations with larger geometries are computationally intensive, we adopted a 2D eigenmode approximation for the enlarged aperture size. Fig. S16 compares the Q-factor distributions from the original 3D simulation (smaller aperture) and the new 2D simulation (larger aperture). The comparison in Fig. S16 shows that the overall Q-factor trends are preserved across scales. The O-VCSEL continues to exhibit a steeply decaying Q-profile dominated by a few high-Q WGMs, while the other cavities display broader and more uniform Q-factor distributions that support multimode lasing. These consistent results confirm that the cavity geometry, rather than aperture size alone, governs the observed Q-factor characteristics.

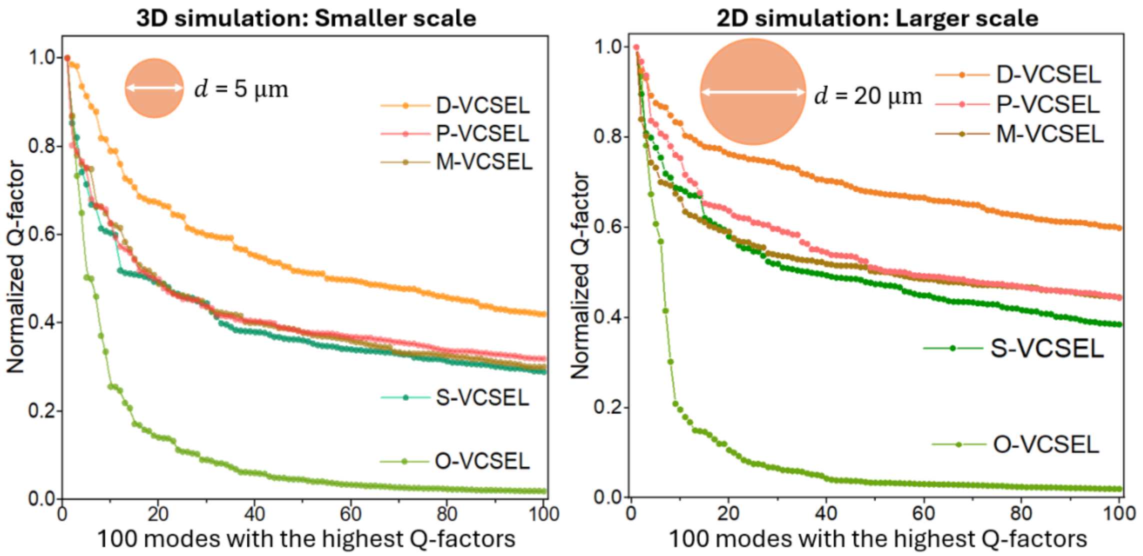

Fig. S16. Normalized Q-factor distributions for the top 100 optical modes of D-, P-, M-, S-, and O-VCSELs, obtained from eigenmode simulations using a reduced aperture diameter of 5 μm (Left) and a larger aperture diameter of 20 μm (Right).

The modal behavior of VCSELs with various cavity geometries was analyzed by comparing simulated fundamental modes, simulated modes with the highest Q-factors, and experimental near-field patterns, as shown in Fig. S17. The fundamental modes, derived from simulation, exhibit smooth intensity distributions in the cavity. These modes represent the basic optical field supported by the cavities, with approximately Gaussian profiles. The modes with the highest Q-factors highlight the spatial confinement of light within the cavity boundaries. In O-, D-, and P-VCSEL, these modes are primarily localized along the edges, especially the curved boundary. The experimental NFPs (bottom row) exhibit more complex intensity distributions compared to the simulated results. These discrepancies arise from practical factors, including non-uniform current injection, thermal effects, and mode competition, which are not accounted for in the idealized simulations. For example, in the M-VCSEL and S-VCSEL, the experimental patterns reveal intricate intensity features with Fabry-Perot-like patterns. While the simulated modes provide valuable insights into the influence of geometry on optical properties, they do not fully replicate experimental results. The neglect of non-uniform current injection, thermal effects, and polarization dynamics in the simulation leads to deviations between the simulated and experimental NFPs. Future work could incorporate these factors into the simulation framework to achieve greater accuracy.
